# Development and Application of an Atomic Absorption Spectrometry-Based Method to Quantify Magnesium in Leaves of *Dioscorea polystachya*

**DOI:** 10.3390/molecules29010109

**Published:** 2023-12-23

**Authors:** David Krüger, Alexander Weng, Daniel Baecker

**Affiliations:** 1 Department of Pharmaceutical Biology, Institute of Pharmacy, Freie Universität Berlin, Königin-Luise-Straße 2+4, 14195 Berlin, Germany; d.krueger@fu-berlin.de; 2 Department of Pharmaceutical and Medicinal Chemistry, Institute of Pharmacy, Freie Universität Berlin, Königin-Luise-Straße 2+4, 14195 Berlin, Germany

**Keywords:** atomic absorption spectrometry, *Dioscoreaceae*, *Dioscorea polystachya*, extraction, graphite furnace, HR CS AAS, leaf extract, magnesium, method development, yam

## Abstract

The Chinese yam (*Dioscorea polystachya*, DP) is known for the nutritional value of its tuber. Nevertheless, DP also has promising pharmacological properties. Compared with the tuber, the leaves of DP are still very little studied. However, it may be possible to draw conclusions about the plant quality based on the coloration of the leaves. Magnesium, as a component of chlorophyll, seems to play a role. Therefore, the aim of this research work was to develop an atomic absorption spectrometry-based method for the analysis of magnesium (285.2125 nm) in leaf extracts of DP following the graphite furnace sub-technique. The optimization of the pyrolysis and atomization temperatures resulted in 1500 °C and 1800 °C, respectively. The general presence of flavonoids in the extracts was detected and could explain the high pyrolysis temperature due to the potential complexation of magnesium. The elaborated method had linearity in a range of 1–10 µg L^−1^ (R^2^ = 0.9975). The limits of detection and quantification amounted to 0.23 µg L^−1^ and 2.00 µg L^−1^, respectively. The characteristic mass was 0.027 pg, and the recovery was 96.7–102.0%. Finally, the method was applied to extracts prepared from differently colored leaves of DP. Similar magnesium contents were obtained for extracts made of dried and fresh leaves. It is often assumed that the yellowing of the leaves is associated with reduced magnesium content. However, the results indicated that yellow leaves are not due to lower magnesium levels. This stimulates the future analysis of DP leaves considering other essential minerals such as molybdenum or manganese.

## 1. Introduction

The Chinese yam (*Dioscorea polystachya*, DP) is one of over six hundred species of the *Dioscorea* genus and a representative of the *Dioscoreaceae* family [1]. As an edible variety, its root tuber is particularly important for nutrition in tropical and subtropical regions [2]. However, apart from its nutritional value, DP has great pharmaceutical potential, as it has been used for centuries in Traditional Chinese Medicine (TCM) [3]. Its pharmacological effects are manifold, with a particular focus on the improvement of glycemia in patients suffering from diabetes [4].

Various functional compounds, such as polysaccharides, saponins, and flavonoids, among others, are responsible for the effects of DP [3]. Optimal conditions are required for cultivation so that the plant produces enough of these phytochemicals. In addition to temperature and light conditions, the soil is also essential.

An optimal or deficient supply of minerals, which are necessary for the biosynthesis of phytochemicals, can be visibly indicated by the leaves in terms of discolorations [5]. The leaves should, therefore, be taken into account, although the root tuber is the most interesting part of DP considering nutritional physiology.

**Figure 1:**
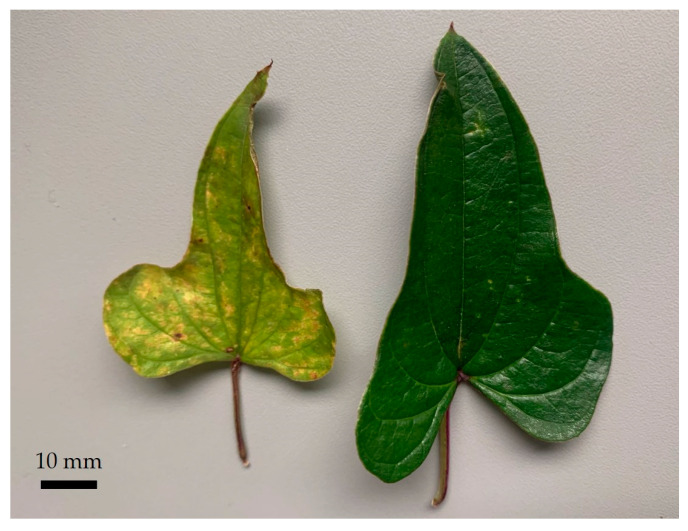
Leaves of *Dioscorea polystachya* showing different degrees of coloration.

In the case of DP, discoloration (Figure 1) can point to a possible undersupply of nutrients to the plant. Such nutrients include, for example, magnesium. It is involved in biochemical and physiological pathways in the plant, such as the synthesis of primary and secondary metabolites [6]. Magnesium is a component of chlorophyll, the green pigment in plant leaves. Thus, a magnesium deficiency can manifest itself in lighter-colored leaves.

The quantification of magnesium is generally feasible, for instance, by using inductively coupled plasma mass spectrometry (ICP MS) and atomic absorption spectrometry (AAS) [7]. Unfortunately, the latter is often inadvertently dismissed as an old-fashioned technique in crisis [8]. Nevertheless, AAS has recently been used in modern pharmaceutical research, for example, in the screening of potential activators of potassium channels [9] or bioanalytical investigations of anticancer and antibacterial drug candidates [10,11]. This documents the usefulness of AAS.

Thus, AAS is still a technique of great value. Moreover, it has several advantages over the competitive technique ICP MS, as summarized previously [12]. The acquisition and operation of an atomic absorption spectrometer is significantly cheaper than an ICP mass spectrometer. This results in a less expensive analysis, although both methods are similarly sensitive. Furthermore, the advancement of AAS into high-resolution continuum-source (HR CS) AAS and molecular absorption spectrometry (MAS) extends its applicability to non-metals such as halogens. While fluorine can hardly be analyzed with commercial argon plasma ICP MS, this is possible with HR CS MAS [12]. Oligopeptides have also been found to be quantifiable using this technique [13]. Given these merits, AAS was chosen for the current study.

When analyzing magnesium using AAS, the fundamental question arises as to the sub-technique. Flame and electrothermal or graphite furnace AAS (F AAS vs. ET AAS/GF AAS) are available. They differ in their manner of atomization, which takes place in a burner flame in the case of F AAS. Electrothermal atomization (heating with electricity) of a graphite furnace is used for GF AAS. While F AAS certainly harbors risks given the use of highly flammable gases, GF AAS requires a smaller sample volume and is even more sensitive [9]. The lower sensitivity of F AAS can be related to the processes taking place in the burner flame. On the one hand, introducing the liquid samples into the flame is prone to interference due to occasional fluctuations in the flow of the burner gas. Moreover, the temperature of the flame is not as constant as in a graphite furnace, which is heated following a consistent temperature program. A higher temperature of the flame may cause additional thermal excitation leading to ionization interference, while a decrease in temperature may be accompanied by insufficient atomization. The latter results in less sensitivity [14] and is generally valid for atoms analyzed using AAS. However, a comparison of atomization using a flame and graphite furnace when analyzing magnesium also showed a higher sensitivity for GF AAS [15]. Consequently, the GF AAS sub-technique was selected for our measurements. The expected concentration range of magnesium in the extracts was not particularly considered when selecting the AAS sub-technique, as employing the more sensitive GF AAS was chosen anyway.

To the best of our knowledge, leaf extracts of DP have not yet been analyzed for some of their biologically relevant metals (e.g., magnesium) using GF AAS. Therefore, the aim of this work was to develop, optimize, and apply an AAS-based method for such an endeavor. This contributes to a currently underrepresented analysis of ingredients in DP leaves, as DP appears to be very promising in terms of its pharmacological profile.

## 2. Results and Discussion

### 2.1. Extraction and Thin-Layer Chromatography

The extracts were prepared using leaves with normal green coloration on the one hand and leaves with less green coloration on the other. In each case, fresh and dried leaves were used for the extraction.

**Figure 2:**
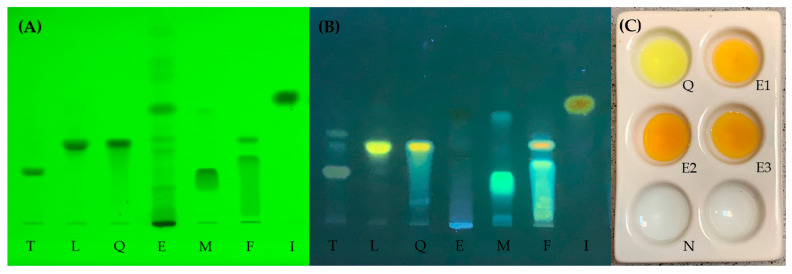
Chromatogram of a leaf extract (E) of *Dioscorea polystachya* using taxifolin (T), luteolin (L), quercetin (Q), morin (M), fisetin (F), and isoliquiritigenin (I) as references detected at (**A**) 254 nm and (**B**) 366 nm after spraying with 2-aminoethoxy diphenyl borate. (**C**) Results of the color reaction for the detection of flavonoids in three leaf extracts (E1–E3) of *Dioscorea polystachya*. A methanolic solution of quercetin (Q) served as a positive control; the extracting solvent nitric acid (N, 12% *w*/*v*) was used as a negative control.

As leaves contain flavonoids, the prepared extracts were routinely analyzed for the presence of flavonoids. For this reason, thin-layer chromatography (TLC) was carried out. Several basic flavonoid compounds, such as rutin (glycoside of quercetin), which has also been found in the leaves of DP before [16], were selected as references. The detection was performed under UV light at 254 nm (Figure 2A) and via fluorescence at 366 nm after spraying with 2-aminoethoxy diphenyl borate (Figure 2B), proving the general presence of flavonoids. In addition, a color reaction was carried out to obtain certainty about the presence of flavonoids in the extracts. In this test, the appearance of a yellowish color also confirmed the presence of flavonoids (Figure 2C).

### 2.2. Selection of the Wavelength

The principle of AAS is based on measuring the attenuation of the intensity of a light beam at a specific wavelength. At this wavelength, the element in question shows a characteristic absorption. Therefore, the selection of the wavelength depends on the analyte. Elements often have different wavelengths of absorption with different sensitivities.

**Figure 3:**
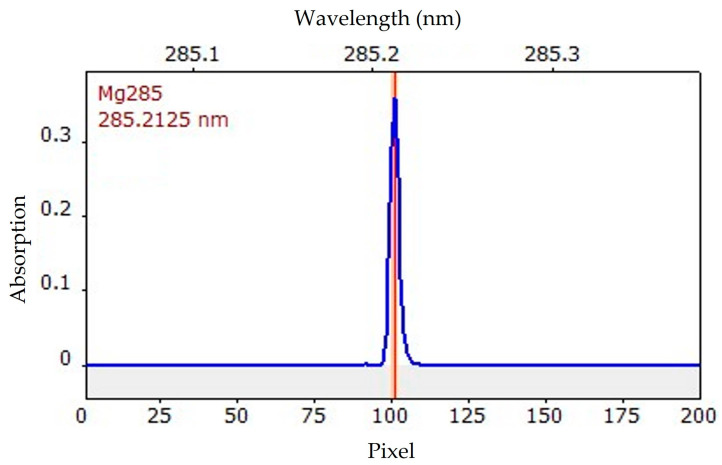
Wavelength-resolved absorption spectrum (blue) of magnesium. The red line represents the primary wavelength of magnesium at 285.2125 nm. The orange bar covers the range of three pixels as used throughout the measurements. This particular spectrum was obtained when quantifying the calibration standard of 5 µg L^−1^.

In the case of magnesium, analysis is possible at a wavelength of 202.582 nm. However, it has a relative sensitivity of only 4.3%. It is considered a secondary line, as a primary wavelength (285.2125 nm) with 100% sensitivity also exists [17]. Therefore, the latter was used throughout the study for the analysis of magnesium. An isolated line appears in the wavelength-resolved absorption spectrum (Figure 3). There are no other lines in its vicinity, which rules out possible interference during the measurement.

### 2.3. Optimization of the Graphite Furnace Program

For the optimization of the time–temperature program, three independently prepared extracts of leaves of DP were used. This was so we could carry out the method development directly with the samples (and their matrices) to be analyzed later. For this purpose, the extracts were diluted with ultrapure water (1:10^6^). The optimization was based on preliminarily designed graphite furnace programs inspired by similar studies [18,19].

In ET AAS, atomization is performed at high temperatures in a graphite furnace. Before this process, however, accompanying compounds in the matrix that could interfere are removed via pyrolysis. The temperatures of these two operating steps are thus essential parameters for a proper analysis. Consequently, the development of a GF AAS method includes the improvement of the pyrolysis and atomization temperatures. In this respect, the approach pioneered by Welz was used [20].

In short, when optimizing the pyrolysis temperature, the atomization temperature was kept constant. The pyrolysis temperature was raised in steps of 100 °C (magnesium: from 900 °C to 1600 °C). A sharp drop in the absorption signal appeared as soon as atomization also took place in the pyrolysis step, and the analyte was, therefore, lost beforehand. The best pyrolysis temperature is the highest temperature (removal of the most accompanying compounds) at which the absorption value does not yet decrease. In a second approach, the pyrolysis temperature was held fixed to adjust the atomization temperature. The latter was then also gradually increased (magnesium: from 1500 °C to 2100 °C) until the increment caused no further enhancement of the absorption signal. The optimum atomization temperature is the lowest temperature (protection of graphite tubes and furnaces) with the highest absorption value.

**Figure 4:**
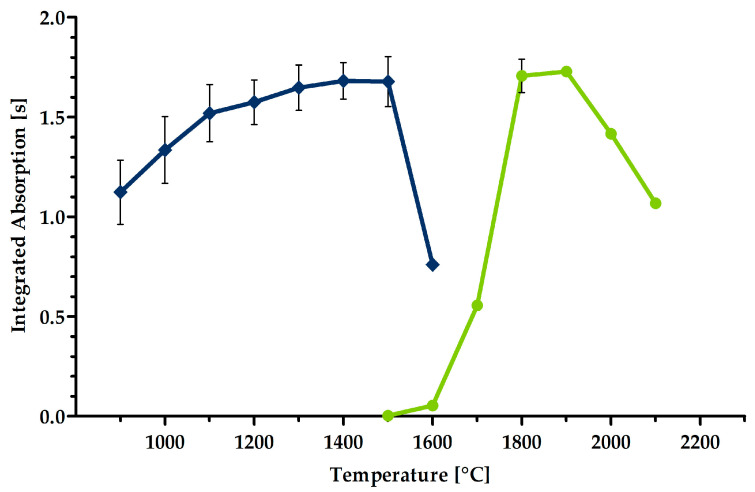
Pyrolysis (blue, left) and atomization (green, right) curves for the analysis of magnesium in leaf extracts of *Dioscorea polystachya*. Extracts made from normal-colored leaves diluted with ultrapure water (1:10^6^) were used (injection volume: 10 µL). The data represent the mean ± standard deviation of *n* = 3 independent replications.

The pyrolysis and atomization curves for the analysis of magnesium are shown in Figure 4.

Although the pyrolysis curve was recorded with the optimized atomization temperature (see below), an increase in the absorption value can be seen when the pyrolysis temperature is elevated. The signals increase by about 50% from 900 °C to 1500 °C. As the pyrolysis temperature advances, organic compounds are progressively removed, which, otherwise, gives rise to a lower signal. That implies the presence of compounds in the matrix that, in some way, mask the analyte magnesium.

As demonstrated with TLC and the identification reaction (see above, Figure 2), the extracts contain flavonoids. Flavonoids are natural compounds that can be structurally assigned to the polyphenol group. It is well known that divalent metal cations such as magnesium can form chelates with flavonoids [21,22,23]. Thus, it can be hypothesized that such complexation may result in the insufficient capture of magnesium if flavonoids are still present in the sample (i.e., a low pyrolysis temperature). However, it was not the aim of the current study to further investigate this assumption for verification.

The optimal pyrolysis temperature for the analysis of magnesium in the current extracts was found to be 1500 °C. At a higher temperature, partial atomization occurred already in the pyrolysis step, so loss of analyte diminished the signal.

In the process of adjusting the atomization temperature, the curve was as expected. As the temperature rose, the signal increased and reached a maximum value. The optimal atomization temperature was set at 1800 °C. A decrease in the signal at temperatures higher than 2000 °C is in agreement with, for example, a case involving quantifying magnesium with GF AAS in desalted crude oil [24].

In addition to these two temperatures, the drying steps were also adapted beforehand. With the help of the equipped furnace camera, it was possible to monitor the samples injected into the graphite tube. The temperatures and heating rates were selected in such a manner that the aqueous samples were dried uniformly without bubble formation and boiling distortion. To ensure this, drying was carried out in three steps. First, the samples were heated to 90 °C (rate: 10 °C s^−1^, holding time: 10 s), then to 100 °C (rate 5 °C s^−1^, holding time: 10 s), and finally to 120 °C (rate: 5 °C s^−1^, holding time: 15 s). The selection of holding times refers to an injection volume of 10 µL, as was used throughout this study. Larger volumes probably require longer times.

Various modifiers are usually investigated to optimize furnace programs. However, as nitrates in general (e.g., deriving from nitric acid used for extraction) and magnesium in particular are universal modifiers [25], the analysis of magnesium is usually carried out without additional modifiers. For this reason, supplementing with modifiers was not performed in the current study.

In summary, the development of a time–temperature program for analyzing magnesium in extracts revealed that the best pyrolysis and vaporization temperatures are 1500 °C and 1800 °C, respectively. These parameters are in a comparable range to similar analyses of magnesium using GF AAS [18,24]. The elaborated graphite furnace program is provided in Table 1.

### 2.4. Calibration and Figures of Merit

For calibration, a magnesium solution for GF AAS was used and diluted accordingly with ultrapure water to prepare the standards. In addition to the blank (ultrapure water only), a total of ten standards (1, 2, 3, 4, 5, 6, 7, 8, 9, 10 µg L^−1^) were used to generate the calibration curve. This concentration range is comparatively high for working with AAS but was intentionally chosen to cover the range of diluted extracts (dilution 1:10^6^; see above). Nevertheless, a linear correlation was ensured for the relatively high absorption values. The squared correlation coefficient, R^2^, had a sufficient value of 0.9975. This shows that even samples that cause high absorption values can be quantified. Consequently, the practical work of sample preparation was facilitated by saving additional dilution steps. On the other hand, the absorption values (higher than actually expected) may indicate well-optimized pyrolysis and atomization temperatures.

The limit of detection (LOD) and the limit of quantification (LOQ) of the elaborated method were determined. For this purpose, the blank (ultrapure water only) was measured eleven times. The mean value of the absorption values ($\bar{x}$) and their respective standard deviations (SDs) were used to calculate the two parameters. The calculation was made according to Equations (1) and (2):

(1) $$LOD=\bar{x}+3\cdot SD$$

(2) $$LOQ=\bar{x}+10\cdot SD$$

The LOD is calculated from the mean value augmented by three times the SD. The LOQ is the mean plus ten times the SD. The LOD amounted to 0.23 µg L^−1^ for the optimized method for the quantification of magnesium. The LOQ was 2.00 µg L^−1^. These particular performance characteristics exceed the values reported in the literature, e.g., for the determination of magnesium using AAS in leaf samples of different origins [26,27,28,29].

Another analytical parameter commonly used in AAS is the so-called characteristic mass ($m_{0}).$ It is expressed as the analyte mass that results in an integrated absorption of 0.0044 s. It can be calculated using data from a calibration standard according to Equation (3):

(3) $$m_{0}=\frac{injection\;volume\;\left(µL\right)\cdot concentration\;\left(µg\;L^{-1}\right)\cdot 0.0044\;s}{integrated\;absorption\;(s)}$$

For example, following this equation provided a characteristic mass $m_{0}$ of 0.027 pg when using a calibration standard with a concentration of 1 µg L^−1^. This value exceeds the one obtained in the quantification of magnesium via GF AAS in desalted crude oil [24] and thus documents the competitiveness of the current method.

The recovery describes the percentage of the analyte concentration found in relation to the specified value. To determine the recovery of the elaborated method, a magnesium standard solution (different from the one applied for the calibration) was used.

This solution (certified concentration: 1003 µg mL^−1^, considered 1 g L^−1^) was appropriately diluted with ultrapure water to obtain solutions with concentrations of 3 µg L^−1^, 6 µg L^−1^, and 9 µg L^−1^. These three concentrations were selected so that they fulfilled the LOQ (2.00 µg L^−1^) on the one hand and covered the lower, middle, and upper range of the linear working range (1–10 µg L^−1^) on the other. The recoveries amounted to 99.9%, 102.0%, and 96.7%, respectively. Consequently, an accurate quantification seems to be possible with this method.

The precision (three injections/measurements of each concentration) was expressed as the relative standard deviation. It was unexpectedly high in the case of 3 µg L^−1^ (11.9%). However, satisfactory values were achieved for the other two concentrations (6 µg L^−1^: 3.1% and 9 µg L^−1^: 2.2%).

The figures of merit of the developed atomic absorption spectrometry-based method used to quantify magnesium are summarized in Table 2.

### 2.5. Quantification of the Magnesium Content in Biological Samples

The developed AAS-based method was applied to determine the magnesium content in the leaves of DP. This is of interest as a lack of magnesium affects the capacity for photosynthesis and also the biochemical pathways of plants, such as the biosynthesis of phytochemicals. A magnesium deficiency may become evident through discoloration of the leaves [30].

Therefore, extracts were prepared from normal-colored leaves (NLs) and discolored leaves (DLs). For each of these two leaf types, extracts were prepared both with dried leaves (DEs) and with fresh leaves (FEs). The elaborated method was applied to two DEs of each NL and DL. Because of a shortage of fresh leaves, it was only possible to examine one FE for each of the NLs and DLs.

All these extracts were quantified for their magnesium content using HR GF AAS. Taking into account the mass of leaves used to prepare the extract, the magnesium content can be expressed as the mass of magnesium (in g) per the mass of leaves (in kg), as summarized in Table 3.

The analysis revealed that the healthy dry leaves (NL-DE) had an average magnesium content of 7.61 g kg^−1^. The content amounted to 7.42 g kg^−1^ in the case of healthy fresh leaves (NL-FE). This indicates that there was no difference regardless of whether fresh or dry leaves were used for the extracts. These values are in good agreement with the range of magnesium quantified via ET AAS in mint tea leaves purchased at marketplaces, for example [19]. Moreover, the obtained values confirm that DP is a significant source of minerals such as magnesium [2].

The discolored dry leaves (DL-DE) had a mean of 20.75 g kg^−1^ magnesium while using fresh leaves (DL-FE) resulted in 19.28 g kg^−1^. Again, no difference was found between the two extraction materials. However, unexpectedly, the discolored leaves showed higher magnesium content than the normal-colored leaves. Different magnesium content in the soil can most likely be ruled out, as the leaves came from plants that grew very close together on the same ground. The soil was not analyzed as part of this study and could, therefore, be regarded as a limitation.

Another mineral that is crucial for plants is molybdenum, as it contributes to protein metabolism, among other things [31]. Its deficiency leads to an accumulation of nitrate. The latter cannot be reduced to amines by molybdenum-requiring enzymes (molybdoenzymes). Amines are necessary for the formation of amino acids. As a result, chlorophyll synthesis is also impaired [32]. This can lead to the discoloration of leaves, too.

A lack of manganese can also cause yellow-colored leaves. Manganese is involved in a metalloenzyme complex during photosynthesis (photosystem II). A deficiency in this manganese-containing cluster may result in reduced photosynthetic processes [33]. Interestingly, manganese may be substituted by magnesium in plants, thus indicating a connection [34]. This encourages more in-depth analysis in future projects.

Consequently, in an upcoming study, the leaf extracts of DP should be investigated in more detail and also with regard to other essential metals such as molybdenum and manganese. Suitable AAS-based methods would also have to be developed.

## 3. Materials and Methods

### 3.1. Chemicals

The solvents and chemicals used in this study were purchased from Carl Roth (Karlsruhe, Germany), PhytoLab (Vestenbergsgreuth, Germany), Sigma Chemical Company (St. Louis, MO, USA), and Merck (Darmstadt, Germany) and employed without further purification. Ultrapure water (Siemens LaboStar, Günzburg, Germany) was used to prepare the calibration standards and to dilute the samples. A commercial magnesium solution for GF AAS (10.0 ± 0.3 g L^−1^) made of magnesium nitrate hexahydrate in 17% nitric acid (Merck, Darmstadt, Germany) was taken to prepare the standards for calibration. A different magnesium atomic absorption standard solution (1003 µg mL^−1^) in 0.5 N of nitric acid (Acros Organics, Geel, Belgium) was applied to evaluate the recovery. Nitric acid 65% (*w*/*v*) (Merck, Darmstadt, Germany) was diluted with ultrapure water to provide 12% nitric acid (*w*/*v*).

### 3.2. Plant Collection and Extraction

The leaves of DP were obtained from the Andreashof, Überlingen, Germany. The Andreashof is an association accredited for the cultivation of Chinese yam. Given the favorable climate conditions in the region around Lake Constance, an appropriate quality for DP tubers can be assured. However, internal and external quality management is provided by the Andreashof and Demeter (Darmstadt, Germany), respectively. At the Andreashof, the DP plants that provided the leaves used in this study were cultivated close together on the same soil. The plants reached a height of about 3–4 m. The respective leaves were collected via manual picking at eye level. Normal-colored and discolored leaves were collected at the same time (age of each plant: around 5–6 months).

Acidic extracts were prepared from normal-colored and discolored leaves, each with both dry and fresh leaves. The dry ones were pounded into a powder by grinding them with a mortar, followed by extraction. The fresh leaves, in contrast, were cut into small pieces within one day after collection and extracted directly afterward. The cut leaves and the powder were dissolved in a ratio of 1:10 with 12% nitric acid (*w*/*v*) in a flask. The latter were covered with a watch glass and extracted for 2 h in a boiling water bath. The hot mixtures were filtered through round filters, grade 595 (diameter: 70 mm; Schleicher and Schuell, Dasse, Germany). The extracts were stored in low-density polyethylene tissue culture flasks (Greiner Bio-One GmbH, Frickenhausen, Germany) at room temperature and protected from light until further analysis. For quantification, the extracts were diluted to 1:10^6^ with ultrapure water.

### 3.3. Thin-Layer Chromatography and Identification Reaction

The samples were analyzed using high-performance thin-layer chromatography (HPTLC) silica gel 60 F_254_ glass plates (10 × 10 cm; Merck, Darmstadt, Germany). To spot the extracts and the reference compounds (taxifolin, luteolin, quercetin, morin, fisetin, and isoliquiritigenin) onto the HPTLC plates (volume: 6 µL), a semi-automatic application device, Linomat IV (CAMAG, Muttenz, Switzerland) was employed. The plates were developed in an automatic developing chamber, ADC 2 (CAMAG, Muttenz, Switzerland). A mixture of chloroform/acetone/formic acid (75/16.5/8.5, *v*/*v*/*v*) served as the mobile phase. Visualization of the plates was performed via observation under UV light at 254 nm and at 366 nm (fluorescence) in the case of spraying with 2-aminoethoxy diphenyl borate.

Apart from TLC, the presence of flavonoids was checked with an identification reaction. For this purpose, equal volumes of extract and a solution of sodium hydroxide (20%) were mixed in a spot plate. Upon twofold dilution of ultrapure water, a yellowish color occurred if flavonoids were present [35]. Following the deprotonation (alkali conditions due to sodium hydroxide) of the polyphenolic structure of the flavonoids, an augmentation of the mesomeric system occurred, resulting in a yellow color. A negative control and a positive control using the nitric acid used for extraction and a methanolic solution of quercetin, respectively, were performed.

### 3.4. Instrumentation

A high-resolution continuum-source atomic absorption spectrometer (contrAA 700, Analytik Jena, Jena, Germany) was employed for the spectrometric measurements; 99.999% argon (Alphagaz, Air Liquide, Düsseldorf, Germany) was applied as both a purging and protection gas. The Aspect CS software (Analytik Jena, version 2.3.1.0) was utilized to control the instrument.

More specifically, the electrothermal (ET) atomization technique was applied. For this purpose, pyrolytically coated standard graphite tubes were placed in the graphite furnace (GF) of the spectrometer. Transverse heating of these tubes caused atomization in the sense of electrothermics. The aqueous samples to be analyzed were stored in 1.5 mL vials made of polystyrole (Sarstedt, Nürmbrecht, Germany). The samples were injected straight into the tube using the MPE 60 graphite furnace autosampler (Analytik Jena). A xenon short-arc lamp served as a continuous light source (wavelengths of 185–900 nm). A double monochromator with a quartz prism pre-monochromator and an echelle grating monochromator (slit width: 1 mm height, 50 µm width), and a linear charge-coupled device (CCD) array detector provided the high resolution.

### 3.5. HR CS AAS Procedure

The quantification of magnesium was based on the measurement of the integrated absorption at the main absorption lines (wavelength magnesium: λ = 285.2125 nm) using three pixels. Above is the elaborated time–temperature program for the GF AAS (Table 1). The mean integrated absorption of triple injections was used when measuring the biological samples.

## 4. Conclusions

The Chinese yam (*Dioscorea polystachya*, DP) is of great importance because of the nutritional value of its tuber. However, the leaves of this yam have not been characterized much so far regarding their components. Given the traditional use of DP for pharmacological purposes, the leaves also seem to be of interest. In this study, an AAS-based method for the quantification of magnesium in leaf extracts of DP was developed. The main focus was on the optimization of a graphite furnace program. The analytical parameters obtained indicate a competitive method. This method was finally applied to the investigation of magnesium in extracts from differently colored leaves of DP. To the best of our knowledge, this is the first time DP leaves have been analyzed for the mineral magnesium using GF AAS. Even though yellowish leaves did not show lower magnesium levels, as expected, method development was achieved as the aim of this study. In the future, the determination of magnesium content could serve as a quality parameter for the leaves or help to monitor and optimize the agricultural cultivation of Chinese yam in general.

## Figures and Tables

**Table 1:** Graphite furnace program for the determination of magnesium (λ = 285.2125 nm) contained in the extracts of leaves of *Dioscorea polystachya*.

Operation	Temperature (°C)	Heating Rate (°C s^−1^)	Holding Time (s)	Argon Flow
Drying 1	90	10	10	Maximal
Drying 2	100	5	10	Maximal
Drying 3	120	5	15	Maximal
Pyrolysis	1500	150	15	Maximal
Auto-zero	1500	0	5	Stop
Atomization	1800	1500	5	Stop
Cleaning	2200	500	5	Maximal

**Table 2:** Analytical performance parameters of the GF AAS method used to quantify magnesium.

Parameter	Value
Linear working range	1–10 µg L^−1^
Correlation coefficient of calibration (R^2^)	0.9975
LOD	0.23 µg L^−1^
LOQ	2.00 µg L^−1^
$\mathrm{Characteristic}\;\mathrm{mass}\;m_{0}$	0.027 pg
Recovery/precision (3 µg L^−1^)	99.9%/11.9%
Recovery/precision (6 µg L^−1^)	102.0%/3.1%
Recovery/precision (9 µg L^−1^)	96.7%/2.2%

**Table 3:** Magnesium content in extracts of different leaves of *Dioscorea polystachya* expressed as mass of magnesium per mass of leaves (g kg^−1^).

Coloration of Leaves	Extraction Material	Name	Magnesium Content (g kg^−1^)	Mean (g kg^−1^)	Standard Deviation (%)
Normal coloration	Dried leaves	NL-DE 1	7.75	7.61	1.74
		NL-DE 2	7.48		
	Fresh leaves	NL-FE	7.42	-	-
Discoloration	Dried leaves	DL-DE 1	18.54	20.75	10.65
		DL-DE 2	22.96		
	Fresh leaves	DL-FE	19.28	-	-

## Data Availability

The data presented in this study are available in the manuscript.
